# The Saturday Fund and St. John's Hospital

**Published:** 1890-02-01

**Authors:** 


### Fallow Crops.

### THE SATURDAY FUND AND ST. JOHN'S HOSPITAL.

The authorities of St. John's Hospital for Diseases of the Skin, which has enjoyed during recent years a full measure of publicity, and seems even yet capable of commanding attention to its affairs, are entitled to whatever satisfaction they may derive from the announcement that the Board of Delegates of the Hospital Saturday Fund, after considering the report of Messrs. W. H. Pannell and Co., public auditors, the salient portions of which we reproduce in another column, p. 279, have granted their institution an award of £59.

We wish we could congratulate either party; but, in truth, we are weary of this long-sustained effort to rehabilitate the fortunes of a hospital which, in common with many of the lower organisms, shows at once so remarkable tenacity of life and so little capacity for rendering its existence useful. Of this we may be sure, that no more highly organised institution could have survived the ordeal which to St. John's has meant nothing worse than a temporary embarrassment, and one is led to question whether the particular speciality affected by St. John's explains the absence of those signs of abashment which only hardened offenders are supposed to be capable of suppressing.

But if the managers of this institution are remarkable for their calm demeanour under trying circumstances, they are positively exemplary in the modesty of their self-appreciation. So thankful are they for testimony of any description, that they have resolved to print and circulate the report of Messrs. Pannell upon the ground that "the special feature in it is that it demonstrates all the moneys given to the hospital have been properly accounted for." Probably no one is more surprised than Messrs. Pannell at the gratification their report has afforded, and to find that the committee of St. John's Hospital are of opinion that its circulation will add to the credit of the institution.

For a hospital which persists, notwithstanding the protest of its auditor, in keeping the records of its receipts and payments "upon loose sheets of paper," which blunders over its entries of payments so that the same amounts appear twice, renders accounts wherein items cannot be traced and totals cannot be made to agree, which has allowed one of its officials when absent from meals provided, to draw an equivalent in money, and makes returns of work done which show four or eight times the reality—for a hospital which has done all these things to feel so elated by the report, where the several eccentricities are enshrined, as to resolve upon its publication, merely because its managers are acquitted of positive malversation, shows amazing insensibility. Yet it is upon the strength of this very report, furnished at their instance, that the Board of Delegates have made their award. It is possible that the majority allowed themselves to be influenced by the fact that the St. John's Hospital is able to boast a certain popularity among the working classes and draws a large proportion of its funds from them. One of the misstatements upon which Messrs. Pannell remark is that towards the total of £765 10s. 6d. returned to the Saturday Fund as "donations," no less than £717 6s. 6d. was contributed by out-patients, leaving but £48 4s. as the actual amount of donations contributed by the philanthropic public during a period of fifteen months. This portentous sum must be taken as the measure of the estimation in which the institution is held outside its own particular following. The Hospital Saturday delegates have now capped this precious contribution with their grant of £59, and if the St. John's managers were at more loss than they appear to be to find a reason for self-congratulation, they might be recommended to say as Lord Melbourne did of the decoration of the Garter, "What I like is that there is no d——d nonsense of merit about it."

Agricola.

*** Our correspondent "Agricola" has treated this matter jocosely, but the question at issue is very serious, and there can be no question that the Hospital Saturday Fund Council, however well-intentioned, have made a great mistake by their action. We can only repeat that the public interest demands the withdrawal and withholding of all subscriptions from the St. John's Hospital for Diseases of the Skin, unless or until a full, impartial, and thorough public enquiry has been held before a competent tribunal.—Editor, The Hospital.

